# Dichloridobis(2-methoxy­dibenzo[*c*,*e*][1,2]oxaphospho­rine-κ*P*)platinum(II) trichloro­methane solvate

**DOI:** 10.1107/S1600536809006643

**Published:** 2009-02-28

**Authors:** Tamás Holczbauer, György Keglevich, Andrea Kerényi, Mátyás Czugler

**Affiliations:** a Institute of Structural Chemistry, Chemical Research Center, Hungarian Academy of Sciences, Pusztaszeri ut. 59-67, 1025 Budapest, Hungary; b Department of Organic Chemistry and Technology, Budapest University of Technology and Economics, 1521 Budapest, Hungary

## Abstract

The title compound, [PtCl_2_(C_13_H_11_O_2_P)_2_]·CHCl_3_, has a rare PtCl_2_ bridging of two dibenzooxaphospho­rine ligands through the metal atom. The Pt^II^ ion is in a slightly distorted square-planar environment. The trichloro­methane solvent mol­ecule shows rotational disorder (major occupancy is 0.75) and is placed near to the inversion centre at (1/2, 1/2, 0) in channels parallel to the *a* axis. The solvent mol­ecule is linked to the complex mol­ecule *via* inter­molecular bifurcated C—H⋯Cl and C—H⋯O hydrogen bonds. The crystal structure is further stabilized by π–π inter­actions involving the benzene rings, with a centroid–centroid distance of 3.658 (8) Å.

## Related literature

For the synthesis of the title compound and related compounds, see: Keglevich *et al.* (2008 ▶) and references therein. For a related phospho­nite structure, see: Claver *et al.* (2000 ▶). For a description of the Cambridge Structural Database, see: Allen (2002 ▶).
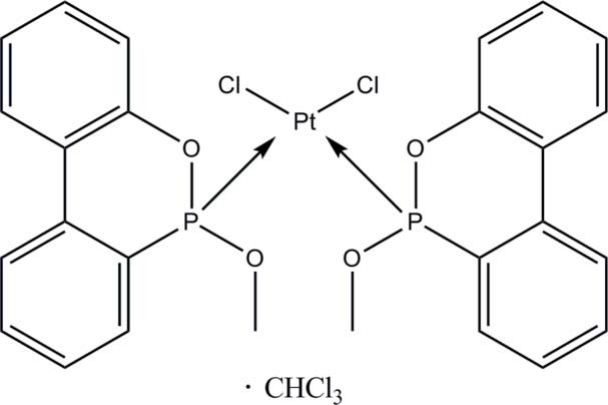


## Experimental

### 

#### Crystal data

[PtCl_2_(C_13_H_11_O_2_P)_2_]·CHCl_3_
*M* *_r_* = 845.73
Triclinic, 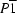

*a* = 10.186 (3) Å
*b* = 13.020 (5) Å
*c* = 13.510 (5) Å
α = 62.402 (11)°
β = 83.128 (12)°
γ = 67.456 (11)°
*V* = 1462.8 (9) Å^3^
*Z* = 2
Mo *K*α radiation
μ = 5.40 mm^−1^
*T* = 93 K
0.40 × 0.30 × 0.15 mm

#### Data collection

Rigaku R-AXIS RAPID diffractometer
Absorption correction: multi-scan (*CrystalClear*; Rigaku, 2008 ▶) *T*_min_ = 0.222, *T*_max_ = 0.498 (expected range = 0.198–0.445)
36911 measured reflections
5339 independent reflections
4822 reflections with *I* > 2σ(*I*)
*R*_int_ = 0.114

#### Refinement

*R*[*F* ^2^ > 2σ(*F* ^2^)] = 0.059
*wR*(*F* ^2^) = 0.151
*S* = 1.06
5339 reflections
381 parameters
66 restraints
H-atom parameters constrained
Δρ_max_ = 3.66 e Å^−3^
Δρ_min_ = −2.69 e Å^−3^

### 

Data collection: *CrystalClear* (Rigaku, 2008 ▶); cell refinement: *CrystalClear*; data reduction: *CrystalClear*; program(s) used to solve structure: *SHELXS97* (Sheldrick, 2008 ▶); program(s) used to refine structure: *SHELXL97* (Sheldrick, 2008 ▶); molecular graphics: *PLATON* (Spek, 2009 ▶); software used to prepare material for publication: *PLATON*.

## Supplementary Material

Crystal structure: contains datablocks I, global. DOI: 10.1107/S1600536809006643/ci2765sup1.cif

Structure factors: contains datablocks I. DOI: 10.1107/S1600536809006643/ci2765Isup2.hkl

Additional supplementary materials:  crystallographic information; 3D view; checkCIF report

## Figures and Tables

**Table 1:** Selected bond lengths (Å)

Pt1—P1	2.188 (3)
Pt1—P2	2.201 (3)
Pt1—Cl1	2.325 (3)
Pt1—Cl2	2.351 (3)

**Table 2:** Hydrogen-bond geometry (Å, °)

*D*—H⋯*A*	*D*—H	H⋯*A*	*D*⋯*A*	*D*—H⋯*A*
C27—H27⋯Cl1^i^	0.97	2.59	3.491 (14)	154
C27—H27⋯O4^i^	0.97	2.57	3.226 (17)	125

Symmetry code: (i) 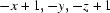.

**Fig. 1.**
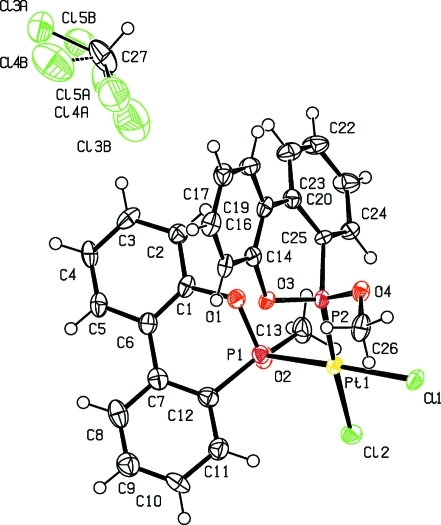
The asymmetric unit of the title compound, with the atomic numbering. Displacement ellipsoids are drawn at the 50% probability level. Suffixes A and B indicate major and minor components of the disordered solvent molecule.

**Fig. 2.**
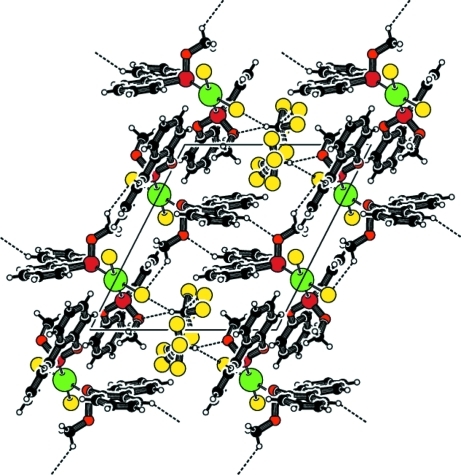
A packing view of the title compound showing intermolecular close contacts as dashed lines.

## supplementary crystallographic information

### Comment

Syntheses, spectroscopic data and theoretical chemistry modeling of various dibenzooxaphosphorines including the parent compound of the title solvate have been reported by Keglevich *et al.* (2008).

The asymmetric unit of the title compound is shown in Fig. 1. The structure of the complex in the crystalline state is in agreement with that reported from theoretical modeling. A more stable *cis* form is prefered over the *trans*, with a *syn*-periplanar disposition of O—P—Pt—P torsion angles (O1—P1—Pt1—P2 *ca* 13.7 (3)° and O3—P2—Pt1—P1 *ca* 49.8 (3)°). However, Pt—P and Pt—-Cl distances appear to be 0.05–0.1 Å shorter in the crystal structure, compared to a theoretical model.

The shortest ring center···center distance of 3.658 (8) Å is observed between C14-C19 benzene rings of complex molecules at (x, y, z) and (1 -*x*, - *y*, 2 - *z*). The only other crystal structure in the CSD (Allen, 2002) having P—Pt—P bridging is MARJEU from the Orpen group (Claver *et al.*, 2000). MARJEU entraps two kinds of solvents in differing stoichiometric ratios (1 for THF and 0.76 for dichloromethane) in its crystal. The title compound also shows disorder of the solvent placed near to inversion centres at (*1/2, 1/2, 0*) in channels parallel to the *a* axis. Partial fixation of the solvent occurs *via* bifurcated C—H ··· O and C—H ··· Cl close contacts from the trichloromethane H atom to an alkoxy O4 atom and to Cl1 (Table 2). Electronic influences of these interactions may also be reflected in the unequal bonding geometry around the metal center. Existence of this C—H···*X* interaction seems to be also corroborated by IR investigations that will be reported elsewhere together with the room-temperature X-ray study of this compound. It appears from these results that the trichloromethane guest disorder is partly of kinetical nature. The C and H atom positions are relatively well kept while chlorine atoms change their positions with respect to the C—H bond.

### Experimental

Synthesis of the parent compound and related ones is reported by Keglevich *et al.* (2008). Recrystallization of the title dibenzo-oxaphosphorine complex from chloroform yielded X-ray quality crystals, solvent content of which became apparent only after the X-ray study.

### Refinement

H atoms were placed in idealized positions (C-H = 0.95-0.98 Å) and refined using a riding model with U_iso_(H) = 1.2-1.5U_eq_(C). The Cl atoms of the trichloromethane solvent molecule were disordered over two sites. The site occupancies and displacement parameters of the disordered atoms were alternately refined and during the final cycles of refinement the occupancies were fixed at 0.75 and 0.25. The C—Cl and Cl···Cl distances involving the disordered atoms were restrained to be equal, and also their U^ij^ parameters were restrained to an approximate isotropic behaviour. The free and restrained refinement models for the solvent resulted in small differences in occupancies of chlorine sites. The restraints virtually improved C—Cl and C···C distances but the maximum positive residual peak appeared close to a minor occupancy Cl site in contrast to the free disorder model, where both maximum and minimum residual densities appeared close to the metal atom.

### Crystal data

**Table:**

[PtCl_2_(C_13_H_11_O_2_P)_2_]·CHCl_3_	*Z* = 2
*M**_r_* = 845.73	*F*(000) = 820
Triclinic, *P*1	*D*_x_ = 1.920 Mg m^−^^3^
Hall symbol: -P 1	Mo *K*α radiation, λ = 0.71070 Å
*a* = 10.186 (3) Å	Cell parameters from 40740 reflections
*b* = 13.020 (5) Å	θ = 3.1–28.7°
*c* = 13.510 (5) Å	µ = 5.40 mm^−^^1^
α = 62.402 (11)°	*T* = 93 K
β = 83.128 (12)°	Prism, colourless
γ = 67.456 (11)°	0.40 × 0.30 × 0.15 mm
*V* = 1462.8 (9) Å^3^	

### Data collection

**Table:**

Rigaku R-AXIS RAPID diffractometer	5339 independent reflections
Radiation source: fine-focus sealed tube	4822 reflections with *I* > 2σ(*I*)
graphite	*R*_int_ = 0.114
Detector resolution: 10 pixels mm^-1^	θ_max_ = 25.4°, θ_min_ = 3.1°
ω scans	*h* = −12→12
Absorption correction: multi-scan (*CrystalClear*; Rigaku, 2008)	*k* = −15→15
*T*_min_ = 0.222, *T*_max_ = 0.498	*l* = −16→16
36911 measured reflections	

### Refinement

**Table:**

Refinement on *F*^2^	Primary atom site location: structure-invariant direct methods
Least-squares matrix: full	Secondary atom site location: difference Fourier map
*R*[*F*^2^ > 2σ(*F*^2^)] = 0.059	Hydrogen site location: inferred from neighbouring sites
*wR*(*F*^2^) = 0.151	H-atom parameters constrained
*S* = 1.06	*w* = 1/[σ^2^(*F*_o_^2^) + (0.0609*P*)^2^ + 24.9249*P*] where *P* = (*F*_o_^2^ + 2*F*_c_^2^)/3
5339 reflections	(Δ/σ)_max_ = 0.001
381 parameters	Δρ_max_ = 3.66 e Å^−^^3^
66 restraints	Δρ_min_ = −2.69 e Å^−^^3^

### Special details

**Table:**

Experimental. Multi-scan empirical absorption correction by T. Higashi in FS-Process

### Fractional atomic coordinates and isotropic or equivalent isotropic displacement parameters (Å^2^)

**Table:**

	*x*	*y*	*z*	*U*_iso_*/*U*_eq_	Occ. (<1)
Pt1	−0.03953 (4)	0.00011 (3)	0.72977 (3)	0.02055 (16)	
Cl1	−0.0887 (3)	−0.1812 (2)	0.8105 (2)	0.0269 (5)	
Cl2	−0.2425 (3)	0.0975 (3)	0.6061 (2)	0.0301 (6)	
P1	−0.0015 (3)	0.1747 (2)	0.6439 (2)	0.0217 (5)	
P2	0.1547 (3)	−0.0974 (2)	0.8424 (2)	0.0206 (5)	
O2	−0.0202 (9)	0.2390 (7)	0.5122 (6)	0.0282 (17)	
O1	0.1586 (8)	0.1516 (6)	0.6736 (6)	0.0244 (15)	
O4	0.1766 (8)	−0.2306 (6)	0.9426 (6)	0.0244 (15)	
O3	0.1682 (7)	−0.0208 (6)	0.9011 (6)	0.0205 (14)	
C8	−0.1302 (14)	0.4864 (11)	0.6809 (10)	0.036 (3)	
H8	−0.0918	0.5447	0.6755	0.043*	
C20	0.4336 (11)	−0.1284 (10)	0.8228 (9)	0.025 (2)	
C5	0.1658 (13)	0.4537 (10)	0.6086 (10)	0.031 (2)	
H5	0.1062	0.5328	0.6039	0.037*	
C6	0.1065 (12)	0.3635 (10)	0.6376 (9)	0.026 (2)	
C4	0.3042 (13)	0.4330 (10)	0.5870 (9)	0.031 (3)	
H4	0.3392	0.4973	0.5683	0.038*	
C3	0.3947 (13)	0.3213 (11)	0.5916 (10)	0.032 (3)	
H3	0.4913	0.3085	0.5751	0.039*	
C2	0.3439 (12)	0.2267 (10)	0.6208 (9)	0.028 (2)	
H2	0.4050	0.1477	0.6261	0.034*	
C1	0.2005 (11)	0.2512 (9)	0.6421 (8)	0.022 (2)	
C12	−0.1048 (12)	0.3029 (10)	0.6709 (9)	0.027 (2)	
C11	−0.2464 (13)	0.3206 (11)	0.6967 (10)	0.032 (3)	
H11	−0.2855	0.2623	0.7034	0.039*	
C10	−0.3290 (13)	0.4235 (10)	0.7126 (10)	0.033 (3)	
H10	−0.4261	0.4379	0.7282	0.040*	
C9	−0.2690 (14)	0.5046 (12)	0.7054 (11)	0.038 (3)	
H9	−0.3249	0.5747	0.7176	0.045*	
C18	0.5477 (11)	−0.1675 (10)	0.9975 (9)	0.027 (2)	
H18	0.6385	−0.2056	0.9761	0.033*	
C19	0.4259 (11)	−0.1218 (10)	0.9284 (9)	0.024 (2)	
C17	0.5387 (13)	−0.1583 (11)	1.0952 (10)	0.031 (2)	
H17	0.6228	−0.1905	1.1409	0.037*	
C16	0.4095 (13)	−0.1031 (11)	1.1274 (10)	0.031 (2)	
H16	0.4044	−0.0966	1.1951	0.038*	
C15	0.2862 (12)	−0.0566 (10)	1.0621 (9)	0.027 (2)	
H15	0.1961	−0.0169	1.0834	0.032*	
C14	0.2973 (11)	−0.0692 (9)	0.9645 (9)	0.023 (2)	
C25	0.3171 (10)	−0.1268 (10)	0.7759 (9)	0.023 (2)	
C24	0.3216 (11)	−0.1342 (10)	0.6771 (9)	0.027 (2)	
H24	0.2402	−0.1315	0.6468	0.032*	
C23	0.4470 (13)	−0.1458 (12)	0.6224 (10)	0.036 (3)	
H23	0.4530	−0.1535	0.5552	0.044*	
C22	0.5620 (12)	−0.1459 (12)	0.6658 (10)	0.035 (3)	
H22	0.6466	−0.1514	0.6270	0.042*	
C7	−0.0446 (12)	0.3848 (10)	0.6641 (9)	0.027 (2)	
C13	0.0389 (14)	0.1694 (12)	0.4507 (11)	0.037 (3)	
H13A	0.1365	0.1117	0.4811	0.055*	
H13B	0.0399	0.2267	0.3718	0.055*	
H13C	−0.0191	0.1218	0.4570	0.055*	
C21	0.5574 (12)	−0.1384 (11)	0.7646 (10)	0.032 (3)	
H21	0.6391	−0.1399	0.7936	0.038*	
C26	0.0861 (14)	−0.2423 (11)	1.0352 (10)	0.035 (3)	
H26A	−0.0134	−0.2095	1.0075	0.053*	
H26B	0.0965	−0.1949	1.0708	0.053*	
H26C	0.1140	−0.3305	1.0901	0.053*	
C27	0.7983 (12)	0.4650 (12)	0.0943 (6)	0.063 (5)	
H27	0.8702	0.3883	0.0978	0.076*	
Cl3A	0.8930 (6)	0.5478 (5)	0.1096 (5)	0.0597 (13)	0.75
Cl4A	0.7076 (9)	0.5499 (5)	−0.0314 (5)	0.096 (3)	0.75
Cl5A	0.7023 (7)	0.4234 (6)	0.2081 (6)	0.092 (2)	0.75
Cl3B	0.6172 (15)	0.478 (2)	0.0820 (17)	0.115 (8)	0.25
Cl4B	0.801 (2)	0.519 (2)	0.1872 (13)	0.101 (7)	0.25
Cl5B	0.8268 (17)	0.5635 (14)	−0.0399 (9)	0.067 (4)	0.25

### Atomic displacement parameters (Å^2^)

**Table:**

	*U*^11^	*U*^22^	*U*^33^	*U*^12^	*U*^13^	*U*^23^
Pt1	0.0207 (2)	0.0197 (2)	0.0229 (2)	−0.01008 (17)	0.00217 (16)	−0.00909 (18)
Cl1	0.0273 (13)	0.0263 (13)	0.0306 (13)	−0.0164 (11)	0.0015 (11)	−0.0105 (11)
Cl2	0.0258 (13)	0.0334 (14)	0.0328 (14)	−0.0082 (11)	−0.0027 (11)	−0.0174 (12)
P1	0.0233 (13)	0.0177 (12)	0.0222 (13)	−0.0071 (10)	0.0002 (10)	−0.0077 (11)
P2	0.0224 (13)	0.0187 (13)	0.0246 (13)	−0.0110 (10)	0.0037 (10)	−0.0107 (11)
O2	0.040 (4)	0.028 (4)	0.022 (4)	−0.016 (3)	0.005 (3)	−0.013 (3)
O1	0.024 (4)	0.017 (3)	0.027 (4)	−0.008 (3)	−0.002 (3)	−0.005 (3)
O4	0.029 (4)	0.019 (4)	0.022 (4)	−0.011 (3)	−0.002 (3)	−0.005 (3)
O3	0.020 (3)	0.026 (4)	0.022 (4)	−0.010 (3)	−0.003 (3)	−0.014 (3)
C8	0.055 (8)	0.023 (6)	0.025 (6)	−0.011 (5)	−0.003 (5)	−0.009 (5)
C20	0.026 (5)	0.020 (5)	0.025 (5)	−0.009 (4)	0.002 (4)	−0.008 (4)
C5	0.041 (7)	0.021 (5)	0.030 (6)	−0.014 (5)	−0.002 (5)	−0.009 (5)
C6	0.032 (6)	0.024 (5)	0.023 (5)	−0.013 (5)	0.002 (4)	−0.009 (4)
C4	0.049 (7)	0.011 (5)	0.027 (6)	−0.014 (5)	−0.001 (5)	0.000 (4)
C3	0.029 (6)	0.041 (7)	0.031 (6)	−0.023 (5)	0.003 (5)	−0.011 (5)
C2	0.028 (6)	0.027 (6)	0.031 (6)	−0.013 (5)	0.006 (5)	−0.014 (5)
C1	0.032 (6)	0.019 (5)	0.014 (5)	−0.015 (4)	−0.003 (4)	0.000 (4)
C12	0.027 (6)	0.024 (5)	0.026 (5)	−0.007 (4)	0.000 (4)	−0.009 (5)
C11	0.033 (6)	0.035 (6)	0.035 (6)	−0.016 (5)	0.005 (5)	−0.020 (5)
C10	0.030 (6)	0.024 (6)	0.032 (6)	0.001 (5)	0.005 (5)	−0.012 (5)
C9	0.039 (7)	0.032 (6)	0.038 (7)	−0.008 (5)	−0.006 (5)	−0.015 (6)
C18	0.021 (5)	0.028 (6)	0.031 (6)	−0.009 (4)	0.002 (4)	−0.012 (5)
C19	0.029 (6)	0.022 (5)	0.023 (5)	−0.016 (4)	0.001 (4)	−0.007 (4)
C17	0.034 (6)	0.027 (6)	0.032 (6)	−0.011 (5)	−0.006 (5)	−0.012 (5)
C16	0.040 (7)	0.033 (6)	0.027 (6)	−0.020 (5)	0.002 (5)	−0.013 (5)
C15	0.037 (6)	0.026 (5)	0.026 (5)	−0.019 (5)	0.002 (5)	−0.013 (5)
C14	0.026 (5)	0.010 (4)	0.023 (5)	−0.008 (4)	−0.002 (4)	0.002 (4)
C25	0.013 (5)	0.024 (5)	0.036 (6)	−0.008 (4)	0.003 (4)	−0.016 (5)
C24	0.020 (5)	0.029 (6)	0.033 (6)	−0.008 (4)	−0.003 (4)	−0.016 (5)
C23	0.029 (6)	0.052 (8)	0.031 (6)	−0.011 (6)	0.007 (5)	−0.026 (6)
C22	0.024 (6)	0.051 (8)	0.034 (6)	−0.014 (5)	0.011 (5)	−0.024 (6)
C7	0.032 (6)	0.021 (5)	0.019 (5)	−0.005 (4)	−0.009 (4)	−0.004 (4)
C13	0.049 (7)	0.039 (7)	0.039 (7)	−0.025 (6)	0.017 (6)	−0.027 (6)
C21	0.023 (5)	0.033 (6)	0.030 (6)	−0.010 (5)	−0.002 (5)	−0.006 (5)
C26	0.052 (8)	0.032 (6)	0.027 (6)	−0.027 (6)	0.006 (5)	−0.010 (5)
C27	0.080 (12)	0.027 (7)	0.061 (10)	−0.004 (7)	0.005 (9)	−0.016 (7)
Cl3A	0.065 (3)	0.056 (3)	0.068 (3)	−0.030 (2)	0.006 (3)	−0.030 (3)
Cl4A	0.136 (6)	0.050 (3)	0.089 (4)	0.004 (3)	−0.058 (4)	−0.038 (3)
Cl5A	0.092 (4)	0.077 (4)	0.126 (6)	−0.049 (4)	0.060 (4)	−0.059 (4)
Cl3B	0.137 (13)	0.104 (11)	0.122 (12)	−0.043 (9)	0.029 (9)	−0.072 (9)
Cl4B	0.106 (11)	0.095 (10)	0.080 (10)	−0.006 (8)	0.001 (8)	−0.047 (8)
Cl5B	0.061 (8)	0.054 (7)	0.068 (8)	−0.015 (6)	0.015 (7)	−0.023 (6)

### Geometric parameters (Å, °)

**Table:**

Pt1—P1	2.188 (3)	C10—C9	1.374 (18)
Pt1—P2	2.201 (3)	C10—H10	0.95
Pt1—Cl1	2.325 (3)	C9—H9	0.95
Pt1—Cl2	2.351 (3)	C18—C17	1.369 (16)
P1—O2	1.575 (8)	C18—C19	1.401 (15)
P1—O1	1.607 (7)	C18—H18	0.95
P1—C12	1.775 (11)	C19—C14	1.376 (15)
P2—O4	1.579 (7)	C17—C16	1.365 (17)
P2—O3	1.584 (7)	C17—H17	0.95
P2—C25	1.780 (10)	C16—C15	1.382 (16)
O2—C13	1.426 (13)	C16—H16	0.95
O1—C1	1.383 (12)	C15—C14	1.389 (15)
O4—C26	1.446 (14)	C15—H15	0.95
O3—C14	1.411 (12)	C25—C24	1.376 (15)
C8—C9	1.366 (18)	C24—C23	1.390 (16)
C8—C7	1.382 (16)	C24—H24	0.95
C8—H8	0.95	C23—C22	1.370 (17)
C20—C21	1.398 (15)	C23—H23	0.95
C20—C25	1.400 (15)	C22—C21	1.378 (17)
C20—C19	1.459 (15)	C22—H22	0.95
C5—C4	1.352 (17)	C13—H13A	0.98
C5—C6	1.399 (15)	C13—H13B	0.98
C5—H5	0.95	C13—H13C	0.98
C6—C1	1.385 (15)	C21—H21	0.95
C6—C7	1.486 (16)	C26—H26A	0.98
C4—C3	1.368 (17)	C26—H26B	0.98
C4—H4	0.95	C26—H26C	0.98
C3—C2	1.389 (16)	C27—Cl4A	1.676 (8)
C3—H3	0.95	C27—Cl5A	1.700 (8)
C2—C1	1.394 (15)	C27—Cl4B	1.705 (9)
C2—H2	0.95	C27—Cl5B	1.738 (9)
C12—C7	1.386 (16)	C27—Cl3A	1.785 (8)
C12—C11	1.398 (16)	C27—Cl3B	1.807 (10)
C11—C10	1.380 (16)	C27—H27	0.97
C11—H11	0.95		
			
P1—Pt1—P2	93.21 (10)	C17—C18—H18	119.3
P1—Pt1—Cl1	176.23 (9)	C19—C18—H18	119.3
P2—Pt1—Cl1	90.20 (9)	C14—C19—C18	116.7 (10)
P1—Pt1—Cl2	88.05 (10)	C14—C19—C20	121.1 (10)
P2—Pt1—Cl2	177.30 (9)	C18—C19—C20	122.2 (10)
Cl1—Pt1—Cl2	88.47 (10)	C16—C17—C18	120.4 (11)
O2—P1—O1	105.7 (4)	C16—C17—H17	119.8
O2—P1—C12	100.9 (5)	C18—C17—H17	119.8
O1—P1—C12	102.7 (5)	C17—C16—C15	120.4 (11)
O2—P1—Pt1	115.3 (3)	C17—C16—H16	119.8
O1—P1—Pt1	110.6 (3)	C15—C16—H16	119.8
C12—P1—Pt1	120.0 (4)	C16—C15—C14	118.3 (11)
O4—P2—O3	103.9 (4)	C16—C15—H15	120.8
O4—P2—C25	102.2 (5)	C14—C15—H15	120.8
O3—P2—C25	103.7 (4)	C19—C14—C15	122.8 (10)
O4—P2—Pt1	117.8 (3)	C19—C14—O3	121.1 (9)
O3—P2—Pt1	112.8 (3)	C15—C14—O3	116.1 (9)
C25—P2—Pt1	114.8 (4)	C24—C25—C20	122.5 (9)
C13—O2—P1	121.6 (8)	C24—C25—P2	120.7 (8)
C1—O1—P1	120.7 (6)	C20—C25—P2	116.4 (8)
C26—O4—P2	119.8 (7)	C25—C24—C23	119.0 (10)
C14—O3—P2	116.7 (6)	C25—C24—H24	120.5
C9—C8—C7	121.3 (12)	C23—C24—H24	120.5
C9—C8—H8	119.4	C22—C23—C24	119.6 (11)
C7—C8—H8	119.4	C22—C23—H23	120.2
C21—C20—C25	116.9 (10)	C24—C23—H23	120.2
C21—C20—C19	122.0 (10)	C23—C22—C21	121.4 (11)
C25—C20—C19	121.2 (9)	C23—C22—H22	119.3
C4—C5—C6	122.8 (11)	C21—C22—H22	119.3
C4—C5—H5	118.6	C8—C7—C12	117.8 (11)
C6—C5—H5	118.6	C8—C7—C6	121.9 (11)
C1—C6—C5	114.8 (10)	C12—C7—C6	120.3 (10)
C1—C6—C7	122.2 (10)	O2—C13—H13A	109.5
C5—C6—C7	122.9 (10)	O2—C13—H13B	109.5
C5—C4—C3	121.2 (10)	H13A—C13—H13B	109.5
C5—C4—H4	119.4	O2—C13—H13C	109.5
C3—C4—H4	119.4	H13A—C13—H13C	109.5
C4—C3—C2	119.3 (11)	H13B—C13—H13C	109.5
C4—C3—H3	120.3	C22—C21—C20	120.6 (11)
C2—C3—H3	120.3	C22—C21—H21	119.7
C3—C2—C1	118.0 (10)	C20—C21—H21	119.7
C3—C2—H2	121.0	O4—C26—H26A	109.5
C1—C2—H2	121.0	O4—C26—H26B	109.5
O1—C1—C6	121.4 (9)	H26A—C26—H26B	109.5
O1—C1—C2	114.8 (9)	O4—C26—H26C	109.5
C6—C1—C2	123.8 (10)	H26A—C26—H26C	109.5
C7—C12—C11	121.1 (10)	H26B—C26—H26C	109.5
C7—C12—P1	119.5 (8)	Cl4A—C27—Cl5A	116.8 (7)
C11—C12—P1	119.4 (9)	Cl4B—C27—Cl5B	111.4 (8)
C10—C11—C12	119.5 (11)	Cl4A—C27—Cl3A	109.2 (6)
C10—C11—H11	120.2	Cl5A—C27—Cl3A	108.1 (5)
C12—C11—H11	120.2	Cl4B—C27—Cl3B	106.4 (7)
C9—C10—C11	119.1 (11)	Cl5B—C27—Cl3B	103.9 (7)
C9—C10—H10	120.4	Cl4A—C27—H27	109.4
C11—C10—H10	120.4	Cl5A—C27—H27	106.7
C8—C9—C10	121.2 (12)	Cl4B—C27—H27	122.5
C8—C9—H9	119.4	Cl5B—C27—H27	96.6
C10—C9—H9	119.4	Cl3A—C27—H27	105.9
C17—C18—C19	121.4 (10)	Cl3B—C27—H27	114.2
			
Cl1—Pt1—P2—O4	−10.8 (3)	C25—C20—C19—C14	−24.2 (15)
Cl2—Pt1—P1—O2	−44.1 (4)	O1—P1—Pt1—P2	13.7 (3)
C12—C7—C6—C1	−13.9 (16)	O3—P2—Pt1—P1	49.8 (3)

### Hydrogen-bond geometry (Å, °)

**Table:**

*D*—H···*A*	*D*—H	H···*A*	*D*···*A*	*D*—H···*A*
C27—H27···Cl1^i^	0.97	2.59	3.491 (14)	154
C27—H27···O4^i^	0.97	2.57	3.226 (17)	125

Symmetry codes: (i) −*x*+1, −*y*, −*z*+1.
